# Molecular and Clinical Insights into *TP53*-Mutated MDS and AML

**DOI:** 10.3390/ijms262210818

**Published:** 2025-11-07

**Authors:** Erotokritos Georgantzinos, Theodoros Karantanos

**Affiliations:** Division of Hematological Malignancies, Sidney Kimmel Comprehensive Cancer Center, Department of Oncology, Johns Hopkins University School of Medicine, Baltimore, MD 21287, USA; erwtokritos19@hotmail.com

**Keywords:** *TP53* mutated MDS/AML, *TP53* mutation, myelodysplastic syndrome, acute myeloid leukemia, clonal hemopoiesis, p53 activation, targeted therapy

## Abstract

*TP53*-mutated myelodysplastic syndrome (MDS) and acute myeloid leukemia (AML) comprise a distinct subgroup of myeloid neoplasms with unique biological and clinical features. The molecular alterations linked to *TP53* mutations drive genomic instability and treatment resistance and ultimately lead to poor survival outcomes. The disease biology is further shaped by alterations in immune response within the bone marrow microenvironment and significant changes in cellular metabolism. Conventional treatments, including chemotherapy and hypomethylating agents +/− venetoclax, offer limited benefit, with high relapse rates and short remissions. Allogeneic bone marrow transplantation is the only curative approach, but the vast majority of patients relapse. Novel therapeutic approaches—ranging from p53 reactivation strategies to immunotherapy and targeted inhibition of specific signaling pathways—are under active investigation. Our review summarizes current knowledge on the molecular pathogenesis, prognostic implications, and therapeutic landscape of *TP53*-mutated MDS/AML and discusses ongoing challenges and opportunities for improving patient outcomes.

## 1. Introduction

P53 is a well-known and broadly investigated tumor suppressor protein with a crucial role in cell cycle regulation, response to DNA damage, and apoptosis [1]. As such, alterations in the *TP53* gene are common across all cancer types and are typically associated with resistance to treatment and adverse prognosis [1,2]. Myelodysplastic syndrome (MDS) and acute myeloid leukemia (AML) are myeloid neoplasms (MNs) caused by somatic mutations in stem and progenitor hematopoietic cells. MDS is thought to be the result of evolving clonal hemopoiesis (CH), and high-risk disease exhibits a high frequency of transformation to AML (secondary AML) [3]. AML is generally characterized by a blast percentage > 20%, but new classification systems propose the MDS/AML entity, with blast count range of 10–20%, which represents a disease continuum rather than two distinct malignancy states [4,5].

*TP53* mutations are present in approximately 10% of patients with MDS and AML and are even more prevalent (70%) in MNs with complex karyotype (CK) [6,7]. The *TP53* gene and its locus on chromosome 17p13.1 can undergo a variety of alternations, but the first event is usually a missense mutation, followed by a bi-allelic loss due to loss of heterozygosity (LOH) and other structural variations. Loss of p53 function promotes genomic instability, resulting in complex chromosomal aberrations and, ultimately, chemoresistance and dismal outcomes for patients [7,8].The underlying mechanism of leukemogenesis is not fully understood, but it has been shown that *TP53* mutations exist in founding clones and are selected under cytotoxic pressure. This also explains the higher prevalence of *TP53* mutations in older patients and in therapy-related myeloid neoplasms (t-MNs) [6,9].

Additionally, in terms of treatment options, intensive chemotherapy is very rarely used, and the addition of Bcl-2 inhibition by venetoclax to hypomethylating agents (HMAs), a combination widely used in older patients ineligible for transplantation, has not demonstrated prominent responses. Allogeneic bone marrow transplantation (Allo-BMT) is the only potentially curative option, yet relapse rates remain high [8]. Moreover, a very small subset of patients achieve the depth of remission required for Allo-BMT. P53-targeting strategies are promising, but developed agents did not exhibit a substantial effect in late-phase clinical trials [3]. Because of this distinctive genomic and clinical profile, *TP53*-mutated MDS/AML has been recently classified separately as a unique disease entity in the World Health Organization (WHO-5) and International Classification Consortium (ICC) systems [4,5].

In this narrative review, we summarize the published data on the molecular profile of *TP53*-mutated MDS/AML and its correlation with clinical outcomes in patients. We also aim to describe current therapeutic strategies and novel agents under investigation in preclinical studies and clinical trials, highlighting the unmet needs in this patient subset.

## 2. Molecular Events and Related Changes in TP53-Mutated MDS/AML

### 2.1. P53 as “The Guardian of the Genome”

P53 tumor protein is a 53-kilodalton protein encoded by the *TP53* gene on the locus of chromosome 17p13.1. Its tertiary structure consists of two N-terminal transactivation functional domains, a central DNA-binding domain (DBD), and a C-terminus encoding its nuclear localization signals and an oligomerization domain for transcriptional activity. P53 plays a critical role in a variety of cellular processes—cell cycle arrest, inflammation, apoptosis, autophagy, metabolism, cell senescence, and genomic stability—through cell growth inhibition in response to DNA damage (“the guardian of the genome”) [1,3]. Recent studies also suggest that abnormal p53 protein can alternate the tumor immune microenvironment, proposing a role in tumor–immune cell interactions [10,11,12].

P53 levels are kept under strict control and regulated by post-transcriptional modifications including ubiquitination, acetylation, and phosphorylation. Mouse double minute-2 homolog (MDM2) is a ubiquitin E3 ligase which drives p53 ubiquitination, leading to proteasomal degradation in the absence of genomic or cellular stress. While MDM2 protein controls p53 stability, MDM4 protein (also called MDMX) inactivates p53 by occluding its transactivation domain. Cellular stress due to hypoxia or DNA damage inhibits MDM2 function, leading to inhibition of p53 ubiquitination and stabilization in its tetrameric form [13]. Activated p53 protein serves primarily as a transcriptional factor with a broad spectrum of target genes involved in almost all cellular processes [1,3].

### 2.2. Alternations of TP53 Gene Locus and p53 Function in Myeloid Neoplasms

The *TP53* gene and its chromosomal locus exhibit substantial heterogeneity of alternations. Most mutations are missense mutations present inside the DNA-binding domain of the protein, followed by insertions/deletions, splice-site mutations, and truncating mutations. Missense mutations affecting the DNA-binding domain result in p53’s misfolding and, thus, an impaired ability to bind to transcriptional targets. P53 can also be inactivated by *TP53* gene deletions, alternative splicing, post-transcriptional modifications, or MDM2-MDM4 upregulation [6]. While TP53-mutated MNs share some common hotspot mutations with other cancers (R175, R248, R273, and R282), these neoplasms are also characterized by unique hotspots (Y220 and M237) [14,15]. In some cases, *TP53* mutations have a dominant-negative effect due to the ability of mutated p53 to form tetramers with the wild-type protein, leading to its inactivation [16]. Nevertheless, missense variants may lead to gain-of-function effects by enabling p53-mutated protein to engage in neomorphic interactions with other transcriptional factors. Besides mutations, loss-of-function can also occur from loss of heterozygosity (LOH) or copy-neutral loss of heterozygosity (cnLOH) [6,7].

In most patients with *TP53*-mutated MDS/AML (76%), there is bi-allelic inactivation of the *TP53* gene. Mechanistically, this is primarily a consequence of ≥2 mutations in 18.4–28.8% of patients, a single mutation with deletion of the trans wild-type allele in 22.5–42.2% of patients, and a single mutation with concomitant cnLOH in 12.2–20.6% of patients. Bi-allelic *TP53* mutations are associated with CK, higher blast count, and higher leukemic transformation [6,17,18].

### 2.3. Concurrent Mutations and Chromosomal Aberrations

In a recent genomic characterization of *TP53*-mutated MNs, whole-genome sequencing (WGS) was performed by analyzing tumor/normal paired samples from 42 patients. In this study, it was shown that mutations in common signaling genes, such as *KIT*, *FLT3*, and *WT1* are very rarely detected, while mutations in epigenetic modifiers (*DNMT3*, *TET2*, or *ASXL1*) or spliceosome genes (*SF3B1*, *SRSF2*) are relatively infrequent (14% and 4.8% cumulative frequency, respectively) [7]. Similar results were published in a previous study including 2200 AML/MDS-with excess blasts (MDS-EB) samples [6]. Indeed, compared with *TP53* wild-type, TP53-mutated MNs are characterized by a paucity of concurrent mutations [15,18].

In the latter study, the majority of patients (84%) with *TP53*-mutated AML/MDS-EB had complex karyotype (i.e., ≥3 concurrent cytogenetic abnormalities) and CK was even more commonly observed in patients with bi-allelic TP53 mutations (97%), multiple *TP53* mutations (94%), and in patients with larger *TP53*-mutated clones (94%, defined by variant allele frequency of >40%) [6]. There is also a reverse relation as follows: *TP53* mutations are observed in 4.5% of cases with normal karyotype, increasing to 17.3% with two chromosomal aberrations and, ultimately, to 76.8% in CK cases. This interrelation is repeated within CK cohorts. In parallel, the paucity of concurrent mutations is more prevalent in multi-hit *TP53*-mutated MNs compared with single-hit [15,18]. These data suggest that even among *TP53*-mutated MNs, multi-hit *TP53* inactivation is a molecularly distinct entity compared with single-hit.

Some additional interesting findings include reduced *ETV6* gene expression in patients with *TP53*-mutated MDS/AML. Possible etiologies include copy-number losses of its chromosomal locus and deletions of the gene, but also unknown mechanisms. Moreover, in *TP53*-mutated AML, *NF1* gene mutations are highly overrepresented, with deletions of one copy of the gene in 45% of cases and bi-allelic mutations in 17%. Furthermore, a surprising finding was that in *TP53*-mutated AML, telomere content was found to be amplified compared to other AML types, with abnormal sequences detected in interstitial chromosomal regions [7].

Regarding chromosomal aberrations, bi-allelic TP53 mutations are strongly linked to complex karyotype (Table 1). In a pool of 42 patient samples analyzed by WGS, recurrent copy-number losses and cytogenetic changes were detected, such as loss of chromosomes 5, 7, 12, 16, 17, 18, and 20q, and gains of chromosomes 21, 22, 1p, and 8 [7]. Furthermore, in myeloid malignancies with *TP53* mutations, chromothripsis—a phenomenon of extensive, randomly oriented chromosomal rearrangements resulting from faulty DNA double-strand break repair—occurs in ~35% of cases [19,20]. This event most frequently involves chromosomes 5, 17, and 21 and ultimately contributes to the formation of complex karyotype [7]. *TP53*-mutated MDS/AML patients with complex cytogenetics have a very poor prognosis, as discussed later in this review.

### 2.4. TP53 Mutations and Changes in Cellular Functions

**Figure 1:**
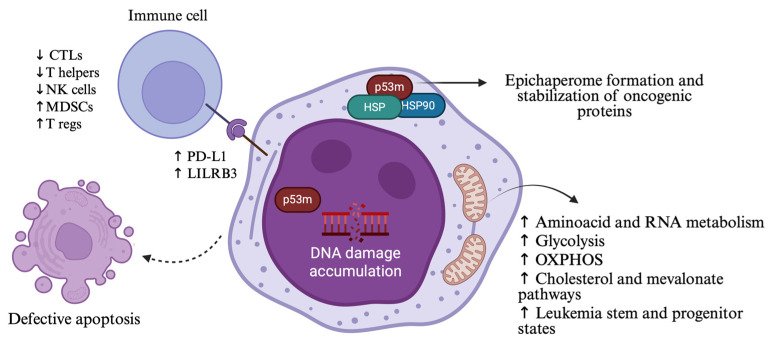
Alternations in cellular functions and immune microenvironment resulting from *TP53* mutations in MDS/AML. Mutant p53 induces genomic instability, impaired apoptosis, immunosuppressive signaling, metabolic rewiring, and formation of epichaperomes that stabilize oncogenic proteins, ultimately contributing to treatment resistance. P53m: mutant p53; HSP: heat shock protein; OXPHOS: oxidative phosphorylation; CTLs: cytotoxic T lymphocytes; NK cells: natural killer cells; MDSCs: myeloid-derived suppressor cells; T regs: T regulatory cells. Created in https://BioRender.com (assessed on 25 September 2025).

P53 regulates a broad spectrum of cellular functions including apoptosis, senescence, glucose and fatty acid metabolism; thus, p53 alternations affect homeostasis beyond genomic instability (Figure 1). In order to assess these effects in cell metabolism, one group performed a multi-omics analysis after developing cell lines with *TP53* mutations (R175H, R273H, and R248Q variants). Elevated expression of gene signatures related to inflammatory responses (*CXCL8*, *HSP8*) and metabolic processes (*ANXA1*, *DUSP1*) was reported in *TP53* R175H-mutated cells, along with enrichment of amino acid and RNA metabolism pathways [21]. Interestingly, our laboratory recently reported that loss of p53 induces a direct, cell-autonomous upregulation of interferon gamma signaling via upregulation of C-C motif chemokine receptor-like 2 (CCRL2)/JAK2/STAT1, which is associated with venetoclax resistance [22]. These findings are consistent with other studies highlighting an association between *TP53* mutations and activation of inflammatory pathways [23].

In the multi-omics analysis, AML cells harboring *TP53* R175H and R273H mutations displayed enhanced oxidative phosphorylation (OXPHOS) and enrichment of leukemia stem cell populations. Some of their findings, including high senescence and OXPHOS states, were associated with poor clinical outcomes [21]. Besides MDS/AML, a pan-cancer analysis of *TP53* mutations and related metabolic pathways showed that *TP53* plays a key role in glycolysis regulation by suppressing the AKT/mTOR and NF-kB signaling pathways and the expression of related genes, such as *PFKP* and *SLC16A3* [24]. Additionally, in a very recent study investigating metabolic drivers of chemoresistance in *TP53*-mutated AML, it has been shown that *TP53*-mutated AML cells resist cytarabine-induced death through upregulation of one-carbon metabolism, that provides greater antioxidant capacity and better cytarabine-induced reactive oxygen species (ROS) management. Remarkably, while stable *TP53*-mutated AML cells had decreased OXPHOS in this study, cytarabine induced an increase in OXPHOS, and the mevalonate pathway was found to play a significant role in these changes [25]. Upregulation of cholesterol pathway has also been linked to CAR-T cell therapy resistance in *TP53*-mutated AML cells [26].

Moreover, heat shock proteins (HSPs), such as HSP90 and other cochaperones and associated proteins that regulate proteostasis, can form epichaperomes in malignant cells. Epichaperomes are complexes of these proteins that stabilize oncogenic kinases, including FLT3, and transcription factors, such as mutant p53, further contributing to signaling aberrations [27]. These complexes have been found to promote cell growth and survival in *TP53*-mutated AML and AML stem/progenitor cells. PU-H71, an epichaperome-specific inhibitor that has been tested in this poor-risk disease, suppresses baseline cellular stress responses and induces apoptosis. Preclinical studies revealed that PU-H71 exhibits a synergistic apoptotic effect with the Bcl-2 inhibitor venetoclax, potentially through Mcl-1 downregulation among other mechanisms. In vivo experiments also revealed promising anti-leukemic activity, raising the hope that malignant cells could be targeted independently of specific mutations [28].

Lastly, while p53 is known to regulate cell cycle arrest and apoptosis through a variety of mechanisms, it was recently found that p53 exhibits its role in cell fate also through ferroptosis, a unique form of iron-dependent cell death mediated by lipid peroxidation. Although most data support that wild-type p53 induce ferroptosis, more research can unveil the effects of mutant p53 and how this can be further exploited in cancer treatment [29].

### 2.5. TP53-Mutated Clones and the Driving Force of Cancer Treatments

Clonal hemopoiesis (CH) and, especially, clonal hemopoiesis of indeterminate potential (CHIP) become more prevalent with advancing age and have been linked to an increased risk of hematologic malignancies. The *TP53* gene is among the top five most frequently mutated genes in CHIP, and the affected hematopoietic stem cells (HSCs) may gain a survival and expansion advantage under certain conditions [30]. Inflammation, bone marrow microenvironment, and prior exposure to chemotherapy, radiation, and immunotherapy can facilitate clonal expansion [3]. *TP53*-mutated clones are more commonly found in patients presenting with t-MNs (23–37%) [9,15] and in patients with cancer predisposition syndromes, such as Li–Fraumeni and Schwachman syndromes (mutations in *TP53* and *SBDS* genes, respectively) [31]. t-MNs have worse prognosis than de novo MDS and AML [9].

The improved outcomes of cancer survivors have brought light to late complications of these therapies. Cancer survivors have a 4.7-fold-higher risk of leukemia [9] and the relative risk for t-MDS/AML ranges from 1.5 to over 10 after chemotherapy for solid tumors [32]. CH present at the time of primary cancer treatment is associated with a 10-fold increased risk of developing t-MNs [31]. Among chemotherapeutic agents, alkylating agents and topoisomerase II inhibitors are the main promoters of t-MNs, compared with anti-metabolites and taxanes [33]. Poly(ADP-ribose) polymerase inhibitors (PARPis) have been associated with an increased incidence of MDS/AML, particularly in patients harboring *TP53* CH mutations and often with a short latency after initial exposure [34]. *TP53* mutations were detected in 50–75% of t-MN cases following PARPi, as described in small cohorts [35,36]. t-MNs can also emerge after chimeric antigen receptor (CAR)-T cell therapies with a cumulative incidence of 7.5–9% at three years. Remarkably, *TP53* mutations are present in 44.4–50% of t-MN cases, and most patients present with t-MN in less than one year after CAR-T therapy [37,38]. Potential risk factors include older age, higher MCV, and higher ICANS grade [38]. All data support that clones harboring *TP53* mutations are present prior to CAR-T therapy [37,38]. In AML patients, pre-leukemic stem cells (preLSCs) harboring somatic *TP53* mutations have been identified, proposing that these mutations constitute early leukemogenic events [39].

### 2.6. The Effect of TP53 Mutations in Immune Microenvironment

Within the bone marrow microenvironment, multiple cell types interact to regulate hemopoiesis in response to external stimuli. Inflammation, and specifically chronic inflammation, plays a key role in MDS pathogenesis and leukemic progression [40,41]. Mutations in RNA splicing and epigenetic regulators, common in MDS, trigger a reciprocal inflammatory cycle through innate immunity signaling pathways and NRP3 inflammasome activation [42,43]. HSCs can respond to cytokines, pathogen-associated molecular patterns (PAMPs), and damage-associated molecular patterns (DAMPs), pushing their differentiation pathway toward myeloid lineage [42].

A single-cell multi-omic analysis of hematopoietic stem/progenitor cells (HSPCs) from patients with myeloproliferative neoplasms (MPNs) showed that, under chronic inflammation, *TP53*-mutated HSPCs exhibit a fitness advantage, driving clonal expansion [41]. Another study compared immune features between *TP53*-mutated and *TP53* wild-type MDS/AML. PDL1, an immune checkpoint protein, was found to be overexpressed on the surface of HSCs in *TP53*-mutant patients, while PD-1 expression was reduced. In addition, there was a substantially decreased number of cytotoxic T cells, T helper cells, and NK cells in the bone marrow of *TP53*-mutated patients. In contrast, a highly immunosuppressive microenvironment was identified, enriched in regulatory T cells (Tregs) and myeloid-derived suppressor cells (MDSCs) (Figure 1) [10].

These findings were corroborated in a study of 61 *TP53*-mutated MDS/AML patients treated with the HMA azacitidine +/− the anti-PDL1 antibody durvalumab in the FUSION clinical trial. T-cell exhaustion with associated immunophenotypes [CD3^+^CD8^+^PD1^+^TIM3^−^ and CD3^+^CD8^+^PD1^−^TIM3^+^] was observed in AML patients harboring *TP53* mutations. Remarkably, after one treatment cycle, DNA methylation patterns at specific loci in PD-1, PD-L1, and PD-L2 genes were almost completely reversed between *TP53*-mutated and wild-type samples compared with baseline [12]. Further research is required to determine whether these epigenetic changes upregulate immune checkpoint expression and contribute to treatment resistance.

Lastly, leukocyte immunoglobulin-like receptor B3 (LILRB3), a myeloid immune checkpoint, shows increased expression in the myeloid cell compartment of *TP53*-mutated MDS/AML. LILRB3 overexpression has been linked to endured leukemic cell survival and impaired T-cell cytotoxicity, posing myeloid immune checkpoints as possible therapeutic targets [44]. These special immunological features of *TP53*-mutated MDS/AML have been a fertile ground, but also an obstacle in the usage of immunotherapy for this specific type of disease, as described later.

## 3. From Classification to Clinical Outcomes: Understanding TP53-Mutated MDS/AML

### 3.1. Classification Systems

As outlined in the previous section, MDS, MDS/AML, and AML with *TP53* mutations share unique genetic and clinical features. The latest classification systems for myeloid neoplasms reflect these findings to improve prognosis assessment, clinical trial design, and drug discovery. In the WHO-5 classification of hematolymphoid disorders (2022), *TP53*-mutated MDS (0–19% blast count) requires bi-allelic inactivation—defined as a *TP53* mutation accompanied by either a second mutation or a copy-number loss/cnLOH) [5,8]. However, TP53-mutated AML (>20% blasts) is not distinguished from the other types of AML, despite the substantially poorer prognosis compared to the wild-type [45,46]. The International Consensus of Classification of myelodysplastic syndromes and related entities (2022) introduces the multi-hit status, defined by either two *TP53* mutations (each with VAF > 10%) or one mutation (VAF > 10%) accompanied by the following: (1) a deletion involving the TP53 locus at chromosome 17p.31, (2) VAF > 50%, or (3) copy-neutral LOH at 17p. Notably, a single *TP53* mutation with complex karyotype is regarded as a multi-hit equivalent. The multi-hit status is essential for *TP53*-mutated MDS categorization (0–9% blasts), but for MDS/AML (10–19%) and AML (>20%) a single mutation with VAF > 10% is sufficient. Both systems require a multi-hit *TP53* status for MDS, excluding MDS patients with mono-allelic inactivation or single-hit status [4]. Further analysis of differences and discrepancies between the two classification systems is beyond the purpose of this review. Finally, the European LeukemiaNet (ELN) 2022 recommendations for diagnosis and management of AML in adults classify *TP53*-mutated AML (VAF > 10% irrespective of allelic status) within the adverse-risk group. A hierarchical classification is proposed as follows: AML with mutated *TP53* precedes AML with myelodysplasia-related gene mutations which, in turn, takes precedence over AML with myelodysplasia-related cytogenetic abnormalities [47].

### 3.2. Role of Allelic Status and Variant Allele Frequency of TP53 Mutations in Patient Outcomes

The impact of allelic status (mono-allelic vs. bi-allelic inactivation) on the prognosis of patients harboring *TP53* mutations is still equivocal. In a large study of 3324 MDS patients, 378 carried at least one putative oncogenic *TP53* mutation (VAF ≥ 2%). Patients were grouped into those with mono-allelic *TP53* state and a residual *TP53* wild-type allele, and those with multi-hit state and probably no residual *TP53*. The multi-hit group exhibited worse overall survival (OS) and higher AML transformation rates compared to the mono-allelic group. In addition, these patients were more cytopenic and had higher percentages of bone marrow blasts (median 9% vs. 4%). Median OS was 8.7 months in multi-hit state vs. 2.5 years in the mono-allelic state. Remarkably, mono-allelic patients had outcomes and responses to therapy comparable to wild-type cases (OS of wild-type patients: 3.5 years). TP53 allelic state predicted outcomes independently from the Revised International Prognostic Scoring System (R-IPSS) [18]. Additionally, although less common in AML (29% single-hit vs. 71% multi-hit), patients with *TP53* single-hit disease exhibited longer OS (8 months vs. 1 month, respectively) [17]. *TP53* mutations also predicted dismal outcomes in t-MDS patients irrespective of bone marrow blast percentage, VAF range, allelic status, or prior treatment type [48]. In the study encompassing 2200 AML/MDS-EB patients, this association between *TP53* allelic status and survival was not confirmed [6]. Nevertheless, strong evidence suggests that multi-hit *TP53* status profoundly worsens survival in AML and MDS with elevated blasts (>5%), although multi-hit cases predominate in this patient group [17]. Interestingly, a study has shown that female patients are characterized by higher CR rates possibly due to the lower percentage of the multi-hit state [49]. These findings are consistent with previous data showing that women with AML more often had normal karyotype and lower-risk mutations [50].

To simplify the categorization progress and facilitate risk stratification, a new model was proposed in 2025. The risk stratification was based on a retrospective analysis of 580 patients with MN harboring *TP53* mutations (VAF > 2%) from Mayo Clinic (USA) and the South Australia Health Network between 2002 and 2023. The WHO-4 revised classification system was used. In MDS-LB (<5% blasts) with mono-allelic *TP53* inactivation, the presence of CK worsened prognosis, approximating outcomes of bi-allelic disease. MDS-EB1 (5–9%), MDS-EB2 (10–19%), and AML (>20%) have been shown to have dismal outcomes regardless of allelic status. MDS-EB1/-EB2/AML cases with VAF < 10% (excluded from ICC 2022 classification) and CK exhibited similar survival rates to those with VAF > 10%. Nonetheless, cases with VAF < 10% and without CK are relatively uncommon. In this study and contrary to the previously presented data, allelic status affected outcomes only in MDS-LB (<5% blasts) group, with no impact in high-blast MDS or AML. *TP53*-mutated AML had the worst outcomes. An online risk calculator derived from this model estimates OS at 6, 12, and 24 months based on patient-specific WHO classification, *TP53* mutation VAF, *TP53* allelic status, and the presence of complex karyotype [51].

Regarding mutation types, although available data are limited, prognostic variability among *TP53* variants has been observed across different myeloid neoplasms (MDS, AML, AML with myeloid-related changes, and t-AML). Interestingly, missense mutations, particularly those retaining transcriptional activity, appear to initiate MDS formation, while truncating mutations may drive leukemic transformation and disease progression [52]. *TP53* splice junction mutations are also referred to predict very poor survival [49]. Several models have been proposed for functional classification of TP53 variants, including the Evolutionary Action score (EAp53)—developed in head and neck cancer—and the Relative Fitness Score (RFS), which assesses the selective advantage of each mutation in cancer evolution. In AML, low-risk *TP53* mutations (RFS > −1) were associated with median OS of 12.9 months, versus 5.5 months for high-risk (RFS ≤ −1) variants, further indicating patients who would benefit from intensive treatment approaches [53]. With regard to co-mutations and their role, mutations in *CUX1*, *U2AF1*, *EZH2*, TET2, *CBL,* and *KRAS* genes, which comprise the ‘EPI6 signature’, are associated with inferior OS at 24 months [49].

To conclude, *TP53* allelic status, mutation VAF, complex karyotype, mutation type, and co-mutational background collectively influence prognosis. The multi-hit state strongly correlates with CK, reflecting underlying genomic instability. Although various VAF cutoffs have been proposed [49,54,55], VAF does not independently affect outcomes in multi-hit disease, indicating that bi-allelic *TP53*-inactivated clones dominate disease biology and drive poor prognosis regardless of clone size. Similarly, co-mutational status predicts survival only in single-hit patients [18]. Lastly, although allogeneic bone marrow transplantation (allo-BMT) remains the only putative curative treatment option for TP53-mutated MDS/AML patients, early post-transplant relapses are frequent and survival remains poor. In line with previous studies [49,56,57], a very recent MD Anderson Cancer Center analysis reported that patients with lower *TP53* mutation VAF (<50%) and without complex cytogenetics benefited most from allo-SCT, achieving a 2-year progression-free survival of 60% [55].

## 4. Treatment Armamentarium and Novel Strategies for TP53-Mutated MDS/AML

### 4.1. Hypomethylating Agents (HMAs) and Their Combination with Venetoclax Take Precedence over Intensive Chemotherapy

*TP53* mutations are more common among elderly AML patients and those with prior exposure to various treatments for malignancies, while these patients often present with low performance status or comorbidities which preclude them from undergoing intensive chemotherapy. Although HMAs (azacitidine and decitabine) are considered the preferred frontline regimen for elderly/unfit patients with AML [58], the presence of *TP53* mutations deprives this group of patients of substantial survival benefit [59]. Interestingly, a 10-day decitabine therapeutic regimen showed overall survival rates that were not significantly different between *TP53*-mutated and *TP53* wild-type patients [60]. However, despite that decitabine is considered a mainstay therapy for *TP53*-mutated myeloid neoplasms, it remains a palliative approach for the majority of patients.

Venetoclax is an orally administered FDA-approved selective Bcl-2 inhibitor which is widely used in everyday clinical practice for chronic lymphocytic leukemia (CLL)/small lymphocytic lymphoma (SLL) and AML treatment. Venetoclax is used in combination with HMAs (particularly azacitidine) in AML patients, based also upon the results of the pivotal study VIALE-A, where the combination of two exhibited higher composite response rates [CR + complete remission with incomplete hematologic recovery (CRi)] compared to azacitidine alone (55.3% vs. 0%) [61]. Nonetheless, a post hoc analysis of the VIALE-A trial revealed that the addition of venetoclax to azacitidine do not benefit patients with *TP53* mutations (median OS: 5.5 months vs. 5.4 months with azacitidine and placebo) [62]. Additionally, in a 2021 post hoc analysis of a phase 2 trial, 10-day decitabine plus venetoclax regimen produced lower response rates in patients harboring *TP53* mutations (ORR 66% vs. 89%, *TP53*-mutated vs. *TP53* wild-type disease) and shorter survival (median OS 5.2 vs. 19.4 months, respectively) [63]. Notably, in contrast to the commonly used cytotoxic doses of HMAs and venetoclax, weekly administered low-dose combination of decitabine and venetoclax can provide a comparable median OS of 11.3 months with less myelotoxicity [64].

Furthermore, the absence of intact p53 protein provides these myeloid malignancies with innate chemorefractoriness and, combined with the high-risk profile of patients, it results in limited usage of intensive chemotherapy. This is also due to the low response rates (20–40%) and median survival (5–11 months) that have been reported [6,15,65]. *TP53* mutation VAF has also been reported to potentially affect survival in patients receiving intermediate- or high-dose cytarabine-based regimens. A VAF > 40% reduced median survival to 5 months compared to 18.1 months with VAF < 40% [66]. Liposomal daunorubicin and cytarabine (CPX-351) showed improved OS compared to HMA + venetoclax as a frontline regimen for AML [67], but no improvement was found when compared to classic 7 + 3 chemotherapy in a cohort of younger patients with adverse cytogenetics AML or high-risk MDS [68].

Lastly, HMA and venetoclax combinations have also been studied as bridging therapies for allo-SCT in patients with high-risk MDS or CMML. In this recent study, six patients harbored *TP53* mutations and all of them had complex karyotypes. Among these patients, an ORR of 100% was achieved, but the two-year post-transplant OS was 50% with a higher two-year post-transplant cumulative incidence of relapse (CIR, 75% for TP53-mutated patients vs. 15.4% for TP53 wild-type) [69].

### 4.2. Allogeneic Stem Cell Transplantation (Allo-BMT)

Allo-BMT is generally considered for all patients with MNs harboring *TP53* mutations (Table 2). Median post-transplant survival is reported to be 1.03 years and OS at 1 and 2 years is 51.4% and 35.1%, respectively. OS was comparable between MDS and AML patients. These are data reported in an international study from seven institutions across the United States and Australia, published in 2025, encompassing 134 patients with *TP53*-mutated MNs [70]. These findings are consistent with that from a previous systematic review and meta-analysis including 297 patients with *TP53*-mutated AML from eight studies [71]. *TP53* mutations are predictors of poor prognosis following allo-BMT in patients with MDS [72]. Our group has also reported that *TP53* mutations strongly predict worse outcomes after allo-BMT in MNs [73], and the presence of CK further decreases survival rates [74]. On the other side, CR at day 100 post-allo-BMT and the occurrence of chronic GvHD can improve event-free survival (EFS) and OS [75]. The role of pre-transplant *TP53* mutation clearance by NGS still remains equivocal [76,77]. Taken together, although advanced age, poor PS, infections, and other factors can be hindrances for transplantation, allo-BMT remains the only putatively curative option, and risk models based on all these genetic and clinical parameters could facilitate better selection of eligible patients [78].

Regarding conditioning regimens, there are conflicting data about post-transplant relapse rates between myeloablative (MAC) and reduced-intensity conditioning (RIC) [79,80]. Nonetheless, more recent data show that MAC and RIC regimens exhibit comparable relapse and survival rates [75]. Interestingly, in the aforementioned international study, it has been shown that melphalan-based conditioning is associated with superior relapse-free survival (RFS), but this finding might be restricted in patients with a low blast percentage of <5% prior to allo-BMT [70]. In order to reduce relapse and improve survival, different post-transplant maintenance strategies have been used, but with limited effectiveness. There are several agents investigated in clinical trials, such as azacitidine (NCT04173533, results to be posted), azacitidine and venetoclax (NCT04161885, results to be posted), decitabine and cedazuridine (NCT04980404, results to be posted), and venetoclax added to fludarabine and busulfan (NCT03613532, ongoing trial) [81,82,83,84].

### 4.3. The Role of Immunotherapy in TP53-Mutated MDS and AML

#### 4.3.1. Immune Checkpoint Inhibitors (ICI)

As mentioned above, in *TP53*-mutated MDS and AML, PD-L1 expression is elevated in HSCs, as opposed to PD-1 expression that is reduced, and the cellular microenvironment is highly immunosuppressive. These findings have led to the investigation of immune checkpoint inhibitors, which are commonly used in solid tumors, as potential additional treatments. Nivolumab, a PD-1 inhibitor, has been tested as a combination therapy with azacitidine in a phase II study of 70 relapsed/refractory AML patients and exhibited modest efficacy, with a median OS of six months in the *TP53*-mutated cohort (16/70 patients) [85]. Nivolumab has also been investigated as induction therapy with cytarabine and idarubicin in patients with newly diagnosed AML or high-risk MDS (eight patients with TP53 mutations), and a median OS of 18.54 months was reported [86]. Ipilimumab, a cytotoxic T-lymphocyte-associated antigen-4 or CTLA-4 inhibitor, has been investigated in combination with decitabine in relapsed/refractory (R/R) or treatment-naïve MDS and AML. Although there was no separate analysis for *TP53* mutations, ORR was 52% in the transplant-naïve group [87]. Dual blockade of PD-1 and CTLA-4 was also tested in previously untreated MDS in a clinical trial with recently published results [88]. The investigated combinations were azacitidine–nivolumab, azacitidine–ipilimumab, and azacitidine–ipilimumab–nivolumab. Azacitidine–nivolumab exhibited the best efficacy, with an ORR of 55%, while the triplet-based regimen had the highest toxicity [88]. In addition, durvalumab, a PD-L1 inhibitor, had an acceptable safety profile but did not improve outcomes in combination with azacitidine in a randomized phase II clinical trial [89]. Lastly, there is an ongoing phase I/II clinical trial investigating the combination of the hypomethylating agent guadecitabine with the anti-PD-L1 antibody atezolizumab for R/R MDS or CMML (NCT02935361) [90].

#### 4.3.2. Anti-CD47 Targeting Strategies

CD47 is widely expressed on the surface of various malignant cells and interacts with the signal-regulatory protein (SIRP)-a on phagocytic cells, initiating an antiphagocytic “don’t-eat-me” signal. Magrolimab was the first-in-class monoclonal antibody targeting this molecule. Magrolimab showed promising results combined with azacitidine in early-phase clinical studies, achieving a 40.3% CR/CRi in patients with *TP53*-mutated AML [91]. In a phase Ib/II clinical trial, magrolimab was combined with azacitidine and venetoclax in patients with newly diagnosed or R/R AML ineligible for intensive chemotherapy, and while response rates were high (ORR 74% for *TP53*-mutated AML and 93% for wild-type), 1-year survival for *TP53*-mutated AML was 53% [92]. Despite these encouraging results, phase III clinical studies ENHANCE-2 [93,94] and ENHANCE-3 [95,96] were discontinued due to futility and increased mortality.

Next-generation CD47 blockers have also been developed, such as evorpacept (ALX148). Although preliminary clinical data of evorpacept in combination with azacitidine and venetoclax showed favorable tolerability and potential anti-leukemic activity, the ASPEN-05 phase 1a study never moved forward to phase 2 [97,98]. The ASPEN-02 phase I/II trial, which tested evorpacept with azacitidine in patients with high-risk MDS, was also discontinued [99]. Finally, lemzoparlimab and ligufalimab (AK117) are two novel CD47-targeting antibodies which are red blood cell-sparing. Lemzoparlimab was tested for safety in a phase 1b study with azacitidine and venetoclax, but the study was terminated due to strategic considerations [100,101]. Ligufalimab is being tested with azacitidine in an ongoing phase 2, randomized, double-blind, placebo-controlled, multicenter study for patients with newly diagnosed higher-risk MDS (NCT06196203) [102]. Phase 1 study of this drug revealed CR rate of 48.1% and limited toxicity [103].

#### 4.3.3. Sabatolimab—A TIM-3 Inhibitor

T-cell immunoglobulin and mucin domain 3 (TIM-3) is an immune regulator expressed on immune cells and myeloid leukemic progenitors. By inhibiting TIM-3, sabatolimab facilitates T-cell activation and induction of phagocytosis. This agent was tested in a phase 1b study in combination with HMAs in patients with very high/high-risk MDS (vHR/HR-MDS) and AML. Results reported acceptable safety profile and durable responses even in patients with adverse-risk mutations, including *TP53* mutations [vHR/HR-MDS: ORR: 71.4%, median duration of response (mDOR): 21.5 months, AML: ORR: 53.8%, mDOR: 12.6 months)] [104,105]. Unfortunately, subsequent phase II trials STIMULUS-MDS1 and STIMULUS-MDS2 were discontinued due to failure to reach their primary endpoints [106,107]. The STIMULUS-AML1 study, which investigated sabatolimab combined with azacitidine and venetoclax in patients with newly diagnosed AML, was also discontinued [108]. The STIMULUS-MDS3 study investigating the same combination in 20 vHR/HR-MDS patients reported an ORR of 86.7% in the 800 mg sabatolimab arm [109].

#### 4.3.4. CD123 × CD3 Bi-Specific Antibodies

Flotetuzumab is a CD123 × CD3 dual affinity antibody that works by enhancing the formation of an immunologic synapse between cytotoxic T cells and AML cells, independently of the major histocompatibility complex (MHC) pathway. Based on data showing that *TP53* mutations induce an immune-filtrated tumor microenvironment that promotes resistance to chemotherapy, flotetuzumab was tested in a phase I/II study of 88 adult patients with R/R AML as salvage immunotherapy. The drug exhibited favorable tolerability and a CR rate of 47% among 15 patients with TP53-mutated R/R AML. Median OS was 10.3 months among those who achieved a complete response [23,110]. APVO-436, another novel CD123 × CD3 bi-specific antibody, was well tolerated and exhibited potential anti-leukemic activity alone and in combination with standard-of-care regimens in a phase 1 study [111]. There is an ongoing phase 1b/2 open-label study of this agent in combination with venetoclax and azacitidine in patients with newly diagnosed AML (NCT06634394) [112].

Besides bi-specific antibodies, CD123 is also targeted by other agents, such as tagraxofusp, which is an FDA-approved drug for treatment of Blastic Plasmacytoid Dendritic Cell Neoplasm (BPDCN). Tagraxofusp (TAG) is a recombinant interleukin-3 protein fused to a truncated diphtheria toxin payload and preclinical data have shown that AML cells resistant to TAG were re-sensitized by azacitidine and became more dependent on Bcl-2. A phase 1b study tested TAG and azacitidine with or without venetoclax in AML patients and the triplet combination was tolerable, without further increasing the risk for infections or capillary leak syndrome. Seven out of thirteen patients (54%) with TP53 mutations achieved CR/CRi or morphologic leukemia-free state (MLFS), while median OS was reported as 14 months in the entire cohort [113]. Tagraxofusp is currently investigated in a phase I trial in combination with azacitidine and/or venetoclax for patients with AML, high-risk MDS, or BPDCN (NCT03113643) [114].

#### 4.3.5. Chimeric Antigen Receptor (CAR)-T Cell Therapies

While CAR-T cell therapies have proven highly effective for lymphoid neoplasms such as B-cell lymphomas, they demonstrate limited applicability to AML and myeloid neoplasms because of the lack of tumor-specific targets, high off-target toxicity, and profound myelosuppression. The hostile tumor microenvironment with highly immunosuppressive features is also a strong caveat. CD33, CD123, CLL1, and IL-1RAP are antigens that CAR-T cell therapies have been developed against for treatment of AML patients in various stages and they are being tested in early-phase clinical trials [8,115]. Another novel strategy uses compound CAR (cCAR)-T cells targeting both CD33 and CLL-1 AML antigens, and initial results from a phase 1 clinical trial have shown high efficacy and manageable toxicity [116,117]. Anti-Tim-3/CD123 CAR-T cell therapy is also a compound strategy currently investigated in patients with R/R AML (NCT06125652) [118].

**Figure 2:**
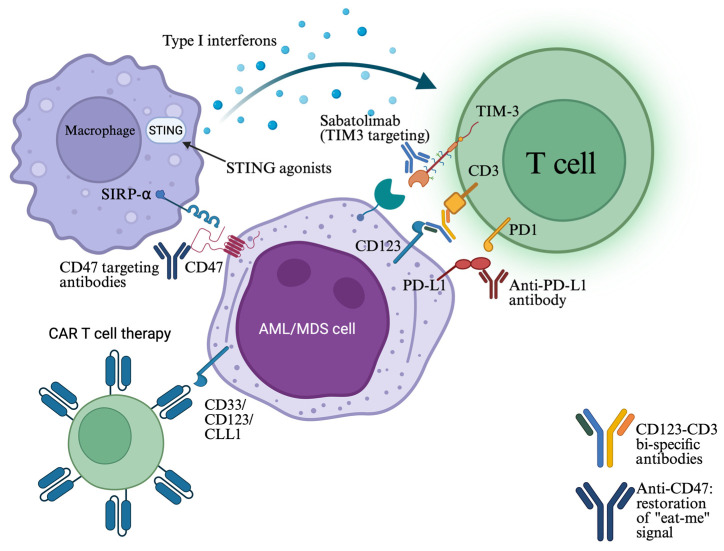
Cell–cell interactions between AML/MDS cells and immune cells, such as T cells and macrophages (phagocytic cells), are crucial in immune escape. Immune checkpoint inhibitors, bi-specific antibodies facilitating T-cell engagement, as well as STING agonists that stimulate innate immune cells, have been developed for restoring immune surveillance and function. CAR-T cells targeting a broad spectrum of antigens are also under investigation (see text). Created in https://BioRender.com (assessed on 25 September 2025).

He et al. [119] developed bi-specific and split CAR (BissCAR) T cells targeting CD13 and TIM3 by isolating multiple nanobodies and using a sequentially tumor-selected antibody and antigen retrieval (STAR) system. In preclinical studies, it has been shown that this platform eradicated patient-derived AML cells with limited toxicity to normal HSCs [119]. Lastly, another group removed CD33 from normal HSCs by using CRISPR-Cas9 gene editing to perform CD33 targeting CAR-T cell therapy while preventing myelotoxicity [120]. Nevertheless, none of the current studies are specifically designed for *TP53*-mutated MDS/AML yet. An overview of different types of immunotherapies currently under investigation for this poor-risk disease is given in Figure 2.

### 4.4. p53 Targeting Strategies

Missense *TP53* mutations cause p53 protein misfolding and loss of function, and these missense mutant proteins are usually overexpressed in malignant cells as a response to oncogenic signals. As a result, refolding the missense mutant protein could induce massive apoptosis to malignant cells with mutated p53 while sparing cells with wild-type protein [121]. Eprenetapopt (APR-246) is the most investigated drug in this category. In two phase II clinical trials testing eprenetapopt in combination with azacitidine, it has been shown that higher ORR (52% and 71%) and CR rates (37% and 44%) can be reached compared to azacitidine alone, and the combination is well tolerated (≥3 cycles given). Additionally, median OS was also extended (12.1 and 10.8 months) and higher CR was associated with *TP53* mutation clearance as detected by NGG with VAF < 5% [122,123]. Unfortunately, in the phase III trial, eprenetapopt plus azacitidine did not reach statistical significance in the primary endpoint (12-month CR rate 35 vs. 22% with azacitidine alone) [124]. Furthermore, eprenetapopt was tested with azacitidine and venetoclax in a phase I trial exhibiting an acceptable safety profile and encouraging activity (ORR: 64%, CR: 38%) [125]. The combination of eprenetapopt plus azacitidine was also evaluated as a post-transplant maintenance therapy in a phase II trial enrolling 33 patients with *TP53*-mutated AML/MDS. The results were promising, with a median RFS of 14.5 months and a median OS of 20.6 months reported [126,127]. Eprenetapopt can also induce cell death via a p53-independent redox effect by inhibiting thioredoxin reductase and thus depleting cellular glutathione levels. Chemoresistance can be acquired by cellular mechanisms exporting glutathione conjugates and that could explain the robust synergistic effects of eprenetapopt with cytotoxic drugs in solid tumors [128].

Arsenic trioxide (ATO), although widely known for its efficacy in acute promyelocytic leukemia in combination with all-trans retinoic acid, has also been found to have mutant p53-rescuing properties [129]. There is a clinical trial testing this drug in *TP53*-mutated MNs (NCT06778187, currently recruiting) [130].

Lastly, while differentiation induction with ATO and ATRA has demonstrated remarkable success in patients with APL, this strategy had not exhibited promising efficacy in other types of AML so far. Very recently, the combination of decitabine with low-dose etoposide showed favorable clinical response in elderly patients with high-risk MDS or AML harboring TP53 mutations compared to TP53 wild-type disease. OS was also improved (31 months in TP53-mutated vs. 9 months in wild-type disease). This effect was confirmed in patient-derived xenograft models, while experiments with TP53 wild-type and deficient AML cell lines revealed a differentiating effect of the combination, resulting in neutrophil terminal differentiation only in TP53-mutant and knockout cells [131]. Table 3 summarizes clinical trials and the outcomes of selected treatment strategies developed for TP53-mutated MDS and AML.

### 4.5. Ongoing Studies of Novel Molecular Targets

#### 4.5.1. Tropomyosin Receptor Kinase (TRK) Inhibition

Recent studies have demonstrated that *TP53* mutations increase the dependency of venetoclax-resistant AML cells on the NTRK pathway [132]. Entrectinib, an NTRK/ALK/ROS1 inhibitor, is currently evaluated in a phase 1 study in combination with oral decitabine and cedazuridine (ASTX727) in patients with *TP53*-mutated R/R AML (NCT05396859). Early results have reported a favorable safety profile, while one patient out of thirteen, who was enrolled following an early post-transplant relapse, achieved a CR lasting for five months [133].

#### 4.5.2. AXL Inhibition

AXL is a tyrosine receptor kinase that is found to be overexpressed in malignant hematopoiesis compared to normal hematopoiesis and has been defined as an adverse prognostic marker in AML patients [134]. Preclinical studies of TP-0903, a multiple kinase inhibitor, have shown anti-leukemic activity in different *TP53*-mutated AML cell lines and prolonged survival in xenograft models, both alone and in combination with decitabine [135]. These findings led to a phase 1b/2 study of this combination as a sub-study of the Beat AML clinical trial (NCT03013998). Among patients harboring TP53 mutations, the overall composite remission rate (CR/CRi/CR with partial hematologic recovery) was 45%, and the median OS was 10 months [136].

#### 4.5.3. Pevonedistat—An NEDD8-Activating Enzyme Inhibitor

Pevonedistat is a first-in-class inhibitor of the NEDD8-activating enzyme and induces apoptosis in AML cells via increased reactive oxygen species production, accumulation of the MYC oncoprotein, and Mcl-1 inhibition through NOXA protein upregulation [137,138]. Although preclinical and early-phase clinical study results were encouraging [137], pevonedistat combined with azacitidine did not eventually improve composite CR rates in older patients with *TP53*-mutated AML [138]. Moreover, the combination of azacitidine, venetoclax, and pevonedistat has been tested in patients with newly diagnosed sAML and with MDS or CMML after failure of HMAs in a phase I/II single-center study. The CR/CRi rate was the same between patients with *TP53*-mutated AML and the entire cohort (64% vs. 66%, respectively), but the median OS was much lower in TP53-mutated AML vs. TP53 wild-type patients (8.1 vs. 18.0 months, respectively) [139].This triplet-based regimen was also investigated in a phase 1 study in patients with R/R AML. Interestingly, a 71.4% CR rate was observed among patients not previously exposed to venetoclax therapy, and CR achievement was associated with extended median OS [140].

#### 4.5.4. PLK4 Inhibition

Recent data revealed that polo-like kinase 4 (PLK4) expression is increased in *TP53*-mutated AML cell lines and primary samples, and its inhibition induces cellular senescence and defective cytokinesis, alternates histone modification, and increases cytokine and chemokine secretion via the cGAS-STING pathway. These effects were found to be mediated by the newly described PLK4/PRMT5/EZH2/H3K27m3 axis, which operated both in *TP53*-mutant and wild-type cells [141]. High PLK4 expression is also associated with dismal prognosis in AML [142]. Based on these findings, CFI-400945, a selective oral PLK4 inhibitor that regulates centriole duplication, was tested in preclinical studies, where it demonstrated antitumor efficacy in xenograft mouse AML models. A phase 1 study tested this drug in patients with very high-risk R/R AML and MDS, and two out of four patients with *TP53*-mutated disease reached CR, while another one had a >50% reduction in bone marrow blasts. Enteritis/colitis was reported as a dose-limiting toxicity [143]. Currently, a newer crystal form of this drug is tested as a single agent or in combination with azacitidine in patients with AML, MDS, or CMML, with preliminary results showing an acceptable safety profile (NCT04730258) [144,145].

#### 4.5.5. STING Agonists

**Figure 3:**
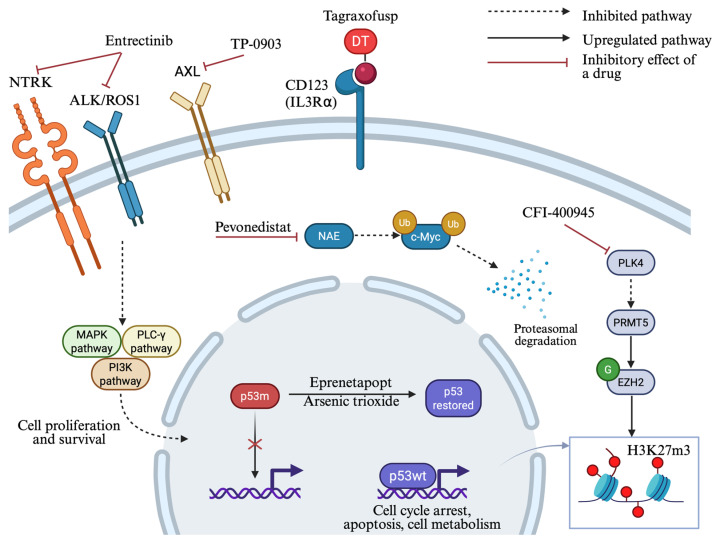
Novel treatment strategies under investigation for *TP53*-mutated MDS/AML. Complex molecular pathways are implicated in *TP53*-mutated disease, giving rise to the development of new targeted agents for this poor-risk subtype. Eprenetapopt and arsenic trioxide (ATO) restore wild-type p53 conformation. Entrectinib inhibits tropomyosin receptor kinases (NTRK/ALK/ROS1), while TP-0903 inhibits the AXL tyrosine kinase receptor, suppressing signaling pathways that promote cell proliferation and survival. Tagraxofusp targets CD123 on malignant cells to induce cytotoxicity through diphtheria toxin release. Pevonedistat inhibits the NEDD8-activating enzyme (NAE), leading to c-Myc accumulation and subsequent NOXA transactivation. PLK4 inhibition by CFI-400945 activates PRMT5, resulting in histone methylation by EZH2 and increased global H3K27me3 levels. DT: truncated diphtheria toxin; p53m: mutant p53; p53wt: wild-type p53; NAE: NEDD8-activating enzyme. Created in https://BioRender.com (assessed on 25 September 2025).

In the immunosuppressive tumor microenvironment of *TP53*-mutated MDS/AML, restoration and enhancement of adaptive immunity through stimulation of innate immune cells may represent an effective therapeutic strategy. Molecules that have an agonistic effect on the stimulator of interferon genes (STING) can induce the production of type 1 IFNs and thus enhance cytotoxic T lymphocyte presence and function around tumor cells (Figure 2) [146]. STING activation induces cytotoxicity in AML cells [147] and GSK3745417, a non-cyclic di-nucleotide (non-CDN) small STING agonist, was evaluated in preclinical studies and exhibited strong cell growth inhibitory effects through apoptosis induction and immune activation [148]. The safety, tolerability, and pharmacologic profile of this drug was investigated in a phase 1 trial encompassing patients with R/R AML and high-risk/very high-risk MDS, but it was terminated early due to financial reasons (NCT05424380, results posted) [149,150]. CRD3874 is another synthetic STING agonist currently in a phase 1 clinical trial (NCT06626633) [151]. Figure 3 summarizes the novel therapeutic approaches against p53 and new molecular targets.

Table 4 summarizes ongoing clinical trials of investigational strategies for *TP53*-mutated MDS/AML.

## 5. Conclusions and Future Directions

*TP53*-mutated MDS/AML is an almost fatal diagnosis, since no significant improvements in therapeutic landscape have been achieved so far. Extensive research in the field continues to reveal mechanisms of treatment resistance, as well as specific molecular characteristics of the disease that could be exploited as leading lights for the development of new treatments. In this review, we aimed to summarize the current knowledge about this poor-prognosis entity and describe the research progress made toward better patient outcomes to the best of our knowledge.

The preexistence of *TP53*-mutated clones as clonal hemopoiesis raises the question of whether biomarkers could be found so that patients with CH could be intensively monitored or preventively treated. This is particularly important since cancer incidence continues to rise and more patients are going to undergo cytotoxic treatments that give a survival advantage to *TP53*-mutated clones. Pharmacological reactivation of p53 is characterized by high complexity due to the many different mutant protein variants and their different levels of functionality, the ability of p53 to be dysfunctional without mutation, and the severe toxicity affecting non-malignant cells. More individualized strategies, such as targeting specific variants, better drug combinations, or implementation of p53-targeting drugs in earlier stages of the disease treatment, could potentially improve their efficacy [122]. Despite the fact that TP53-mutated MDS and AML represent a significant unmet medical need, the majority of ongoing clinical trials are not specifically focused on this molecular subgroup. Therefore, the design and implementation of molecularly defined trials could facilitate improved patient outcomes. Additionally, more in-depth research aimed at identifying biomarkers of response beyond cytogenetics, VAF measurement, or MRD assessment, could guide precise patient selection for each therapeutic modality.

Drug repurposing is also a novel strategy investigated in this group of patients. Mevalonate pathway has been found upregulated in *TP53*-mutated AML and it correlates with mitochondria-dependent chemoresistance and CAR-T cell therapy failure [25,26]. Statins, widely used mevalonate pathway inhibitors, are investigated for this purpose and pitavastatin has been tested in combination with venetoclax in a phase 1 clinical trial, where it showed favorable tolerability with all patients achieving complete response [152]. Niclosamide, an oral anthelminthic medication used to treat tapeworm infections since 1960, has also been tested in vitro in *TP53*-mutated AML patient samples and cell lines, exhibiting potential anti-leukemic activity and restoration of sensitivity of these cells to hypomethylating therapy [153].

Finally, activation and redirection of innate and adaptive immunity into fighting this disease remains a challenge, mainly because of the different immune evasion mechanisms that AML cells use to escape. In-depth research in cancer immunology can unravel these mechanisms and find new treatments. Until better therapies are developed, allo-BMT in first remission should be attempted in all eligible patients.

## Figures and Tables

**Table 1:** Common cytogenetic abnormalities in *TP53*-mutated MDS/AML.

Type of Aberration	Chromosomes Involved	Notes	Reference
Chromosomal loss	5 (del5q), 7, 12, 16, 17 (del17p), 18, and 20q	Recurrent abnormalities in *TP53*-mutated MDS/AML	[6,7]
Chromosomal gain	21, 22, 1p, and 8		[7]
Chromothripsis	5, 17, and 21	35% in TP53-mutated MNs with CK	[7,19]
Complex karyotype	>3 concurrent cytogenetic abnormalities	84% of patients with *TP53*-mutated AML/MDS-EB	[6]

**Table 2:** Clinical outcomes of Allo-BMT in *TP53*-mutated myeloid neoplasms.

Study	Intervention	Outcome
Baranwal, et al. [70]	Allo-BMT in patients with TP53-mutated MNs	Median survival: 1.03 year OS at 3 years: 25.1%
Shahzad, et al. [71]	Systematic review and meta-analysis of 8 studies investigating allo-BMT in TP53-mutated AML	Pooled OS: 21% (median follow-up at 3 years) Pooled RR: 58.9% (at a median of 1.75 years)
Pasca, et al. [74]	Allo-BMT vs. no allo-BMT in patients with TP53-mutated MNs	Median survival 18.9 months vs. 4.1 months, respectively
Senapati, et al. [78]	Allo-BMT vs. no allo-BMT in patients with TP53-mutated AML	Median OS: 13.6 vs. 7.6 months, respectively, Median RFS: 9.3 vs. 4.5 months, respectively

**Table 3:** Selected clinical trials of developed agents targeting TP53-mutated MDS/AML.

Intervention	Phase	Outcome	Trial Identifier
Eprenetapopt (APR-246) combined with venetoclax and azacitidine for the treatment of TP53-mutated MNs	I	ORR: 64%, CR: 38%	NCT04214860
Eprenetapopt (APR-246) ± azacitidine for the treatment of TP53-mutated MDS	III	Not reaching primary endpoint (12-month CR rate 35 vs. 22% with azacitidine alone)	NCT03745716
Magrolimab vs. placebo with azacitidine and venetoclax for patients with untreated AML unfit for intensive therapy (ENHANCE-3 trial, discontinued)	III	Median OS 7.4 months vs. 6.9 months, respectively in the lower-benefit group (including TP53-mutated AML patients)	NCT05079230
Flotetuzumab in Primary Induction Failure (PIF) or Early Relapse (ER) AML (VOYAGE study)	I/II	CR rate: 47% among patients with TP53-mutated R/R AML, median OS: 10.3 months in responding patients	NCT02152956
Tagraxofusp combined with azacitidine ± venetoclax in AML	Ib	54% of patients with TP53 mutations achieved CR/CRi/MLFS	NCT03113643
Sabatolimab combined with azacitidine or decitabine in patients with HR/vHR-MDS and AML	Ib	ORR: 71.4%, mDOR: 21.5 months in patients with HR/vHR-MDS and TP53 mutations	NCT03066648

**Table 4:** Ongoing clinical trials of investigational strategies for *TP53*-mutated MDS/AML.

Intervention	Mechanism of Action	Phase/Status	Trial Identifier
Entrectinib combined with ASTX727 (decitabine and cedazuridine) for the treatment of TP53-mutated R/R AML	NTRK/ALK/ROS1 inhibitor	I—active, not recruiting	NCT05396859
CFI-400945 ± azacitidine in patients with AML, MDS or CMML	PLK4 inhibitor	Ib/II—active, not recruiting	NCT04730258
CRD3874-SI in patients with R/R AML	Synthetic STING agonist	I—active, not recruiting	NCT06626633
Oral-ATO combined with ascorbic acid and investigator choice of low-intensity therapy (HMAs ± venetoclax) for previously untreated or R/R TP53-mutated MDS, AML, or CMML	Mutant p53 reactivation (ATO)	I—active, currently recruiting	NCT06778187
SL-401 (Tagraxofusp) combined with azacitidine ± venetoclax in patients with AML, high-risk MDS, or BPDCN	Recombinant IL-3 protein fused to a truncated diphtheria toxin payload	I—active, currently recruiting	NCT03113643
APVO436 combined with azacitidine and venetoclax in patients with newly diagnosed AML	CD123 × CD3 bi-specific antibody	Ib/II—active, currently recruiting	NCT06634394
AK117 (Ligufalimab) or placebo combined with azacitidine in patients with newly diagnosed high-risk MDS	Next-generation CD47 blocker	II—active, currently recruiting	NCT06196203

## Data Availability

Not applicable.
